# Novel insights into stress-induced susceptibility to influenza: corticosterone impacts interferon-β responses by Mfn2-mediated ubiquitin degradation of MAVS

**DOI:** 10.1038/s41392-020-00238-z

**Published:** 2020-09-18

**Authors:** Zhuo Luo, Li-Fang Liu, Ying-Nan Jiang, Lu-Ping Tang, Wen Li, Shu-Hua Ouyang, Long-Fang Tu, Yan-Ping Wu, Hai-Biao Gong, Chang-Yu Yan, Shan Jiang, Yu-Hui Lu, Tongzheng Liu, Zhenyou Jiang, Hiroshi Kurihara, Yang Yu, Xin-Sheng Yao, Yi-Fang Li, Rong-Rong He

**Affiliations:** 1 Guangdong Engineering Research Center of Chinese Medicine & Disease Susceptibility, Jinan University, Guangzhou, 510632 China; 2 International Cooperative Laboratory of Traditional Chinese Medicine Modernization and Innovative Drug Development of Chinese Ministry of Education (MOE), College of Pharmacy, Jinan University, Guangzhou, 510632 China; 3 Guangdong Province Key Laboratory of Pharmacodynamic Constituents of TCM and New Drugs Research, College of Pharmacy, Jinan University, Guangzhou, 510632 China; 4 School of Traditional Chinese Materia Medica, Shenyang Pharmaceutical University, Shenyang, 110016 China; 5 Institute of Tumor Pharmacology, College of Pharmacy, Jinan University, Guangzhou, 510632 China; 6 Department of Microbiology and Immunology, Basic Medicine College, Jinan University, GuangZhou, 510632 China

**Keywords:** Molecular biology, Innate immunity, Infection

## Abstract

Although stress has been known to increase the susceptibility of pathogen infection, the underlying mechanism remains elusive. In this study, we reported that restraint stress dramatically enhanced the morbidity and mortality of mice infected with the influenza virus (H1N1) and obviously aggravated lung inflammation. Corticosterone (CORT), a main type of glucocorticoids in rodents, was secreted in the plasma of stressed mice. We further found that this stress hormone significantly boosted virus replication by restricting mitochondrial antiviral signaling (MAVS) protein-transduced IFN-β production without affecting its mRNA level, while the deficiency of MAVS abrogated stress/CORT-induced viral susceptibility in mice. Mechanistically, the effect of CORT was mediated by proteasome-dependent degradation of MAVS, thereby resulting in the impediment of MAVS-transduced IFN-β generation in vivo and in vitro. Furthermore, RNA-seq assay results indicated the involvement of Mitofusin 2 (Mfn2) in this process. Gain- and loss-of-function experiments indicated that Mfn2 interacted with MAVS and recruited E3 ligase SYVN1 to promote the polyubiquitination of MAVS. Co-immunoprecipitation experiments clarified an interaction between any two regions of Mfn2 (HR1), MAVS (C-terminal/TM) and SYVN1 (TM). Collectively, our findings define the Mfn2-SYVN1 axis as a new signaling cascade for proteasome-dependent degradation of MAVS and a ‘fine tuning’ of antiviral innate immunity in response to influenza infection under stress.

## Introduction

Influenza viruses are highly contagious diseases and their outbreaks have led to severe causes for death worldwide.^1^ Their high morbidity and mortality rates have resulted in huge economic and social burdens.^2^ However, it is known that not all the people would be infected by influenza virus during its outburst.^3^ Host factors play an important role in determining the outcome of an influenza infection.^4,5^ Pathogen resistance might be one determining factor that effectively eliminates the virus from the body; whereas, another one could be pathogen tolerance, which could reduce or alleviate tissue damages caused by virus infection in the host. A breach of either pathogen pathway can destroy the health balance of host organisms, thereby making them susceptible individuals. It has been reported that the exposure to stress or fatigue would make people more susceptible to influenza infection.^6^ Stressful experiences increase both the susceptibility and frequency of diseases, prolong recovery time, and enhance the incidences of secondary health complications after viral infection.^6,7^ However, the exact mechanism in deciding how stress enhances host susceptibility to influenza viruses remains elusive.

Accumulating evidences have demonstrated the link of stress-provoked disease susceptibility and compromised immune functions.^8,9^ During viral infection, the host’s defense system plays crucial roles in maintaining the fitness dependent on complicated interactions of innate and adaptive immune components. In response to influenza infection, innate immunity is activated to produce type I IFNs (IFN-β) in restricting virus replication and spread.^10^ Stressful experiences could activate the hypothalamic-pituitary-adrenal (HPA) axis, resulting in an elevation of stress hormones, mainly glucocorticoids (GCs) that could induce immunosuppression.^11,12^ Our previous studies have indicated that stress-provoked host’s susceptibility to influenza was associated with impeding IFN-β response.^13,14^ However, how stress influences IFN-β response during influenza virus infection needs to be further dissected.

Among well-defined signals involved in producing IFN-β, mitochondrial antiviral signaling (MAVS) protein-dependent pathway is most significant. Upon recognition of RNA virus by retinoic acid inducible gene-I (RIG-I), MAVS functions as the adaptor to drive the phosphorylation of interferon regulatory factor 3 (IRF3), followed by IFN-β transcription.^15,16^ Intriguingly, stress has been reported to impact the mitochondria function and dynamics, which raised the possibility that stress-inhibited IFN-β response might stem from the deterioration of MAVS antivirus signaling.^17,18^ In this study, we utilized restraint stress mouse model to elucidate the potential mechanism responsible for stress-increased susceptibility to influenza. Our results demonstrated that the increase of corticosterone (CORT), as a result of stress response, hindered IFN-β expression to enhance virus susceptibility. Mechanistically, CORT caused an increased protein level of Mitofusin 2 (Mfn2), which recruited E3 ligase SYVN1 to initiate the degradation of MAVS by proteasome-dependent pathway. Our findings provide novel insights into the mechanisms by which stress induced susceptibility to influenza, and identify CORT pathway as a potential therapeutic target for influenza infection, especially for those patients bearing stress.

## Results

### Stress increases mortality rate and virus replication in influenza-infected mice

**Fig. 1:**
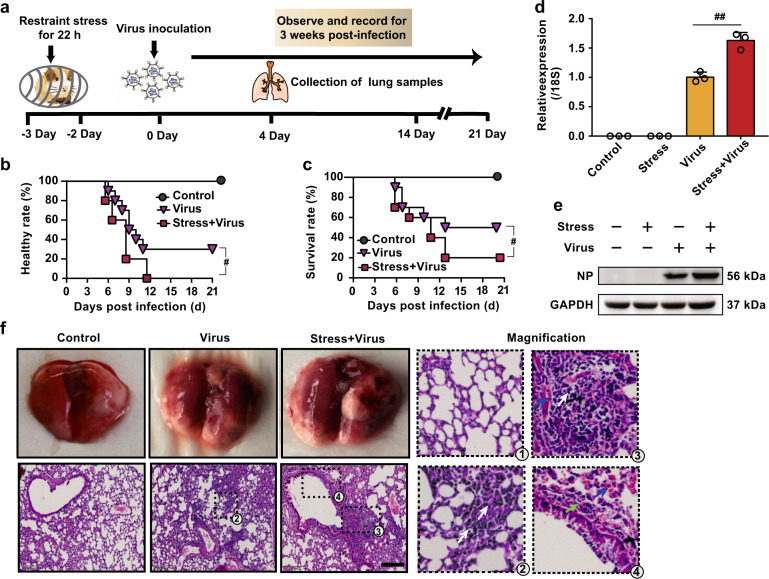
Restraint stress increases the morbidity, mortality, and virus replication in influenza-infected mice. **a** The schematic protocol of animal treatment. Mice were fixed in a restraint cage for 22 h and then recovered for 2 d. H1N1 virus (2×LD_50_) was then administrated nasally to mice. **b**, **c** When mouse showed typical influenza symptoms, including weight loss, hunched back, ruffled fur, altered respiration, and unresponsiveness, as well as decreased body weight (over 1 g/d), it was deemed in morbid state. The morbidity and mortality in each group were examined by recording the percentage of animals remaining healthy or surviving due to H1N1 virus exposure for 21 d or until death (*n* = 10). **d**, **e** On day 4 post virus infection, NP gene and protein expressions were evaluated in the lung tissues by qPCR and western blotting (*n* = 3). **f** Histopathologic changes in the lung tissues examined by H&E staining on day 4 post virus infection. Scale bar, 200 μm. White arrows indicate the presence of inflammatory infiltrates. Green arrows indicate thickened alveolar wall. Blue arrow indicates hemorrhage exudate. CORT, corticosterone. Data were presented as mean ± SD. ^*#*^*P* < 0.05, ^##^*P* < 0.01 vs. Virus group

To determine whether stress increases the susceptibility of mice to influenza virus infection, mice were immobilized in a polypropylene restraint tube for 22 h, and a 2-day recovery was performed before inhalation of influenza virus. The morbidity and mortality of mice were then recorded for 3 weeks (Fig. 1a). Both the morbidity and mortality of stressed mice infected with virus on the 3rd day post restraint were significantly higher than those of the non-stressed/virus-infected mice (Fig. 1b, c), which was consistent with our previous reports.^13,14^

In addition to morbidity and mortality rates, we demonstrated that the gene and protein expressions of viral nuclear protein (NP), an indicator of viral replication level, were more dramatically enhanced in mice infected with virus post-stress than that of non-stressed/virus-infected mice on the 4th post infection (Fig. 1d, e). In the meantime, an obvious lung inflammation was observed in the lungs of virus-infected mice, which was further aggravated in stressed plus virus-exposed mice, as evidenced by thickened alveolar walls, the infiltration of inflammatory cells, and hemorrhage exudates (Fig. 1f). These findings demonstrated that restraint stress enhanced the mortality and viral replication in mice infected with influenza virus.

### CORT is critically involved in stress-provoked influenza virus susceptibility

**Fig. 2:**
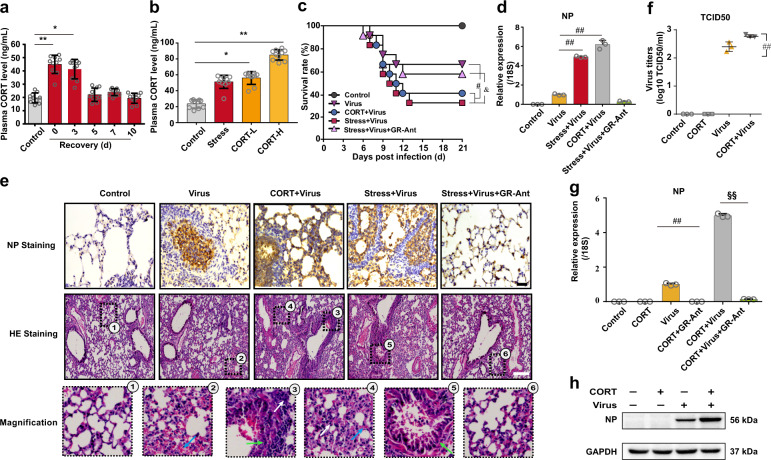
CORT enhances susceptibility to influenza virus in mice. **a**, **b** Plasma CORT concentrations detected by HPLC-UV method in mice at different times post-restraint stress and in mice subcutaneously injected with CORT (1 and 2 mg/kg) for 2 d (*n* = 10). **c** Mice were subcutaneously injected with CORT (1 mg/kg) or GR antagonist RU486 (25 mg/kg) for 2 d, and then inoculated with H1N1 virus. The survival rate of virus-infected mice was monitored for 21 d (*n* = 10). **d** On the 4th day after H1N1 infection, NP gene expression in the lungs tissues were measured by RT-qPCR (*n* = 3). **e** NP protein expression and histological changes were analyzed by immunostaining (scale bar, 50 μm) and H&E staining (scale bar, 200 μm) in the lung sections, respectively. White arrows indicate the presence of inflammatory infiltrates. Green arrows indicate thickened alveolar wall. Blue arrow indicates hemorrhage exudate. **f** A549 cells were pretreated with CORT (100 μM) for 48 h, and then infected with H1N1 virus (10 TCID_50_). The viral titer was determined by TCID_50_ assay at 12 h post infection (*n* = 3). **g**, **h** the expression of NP gene and protein were measured by RT-qPCR and western blotting (*n* = 3). CORT, corticosterone; GR-Ant, glucocorticoids receptor antagonist (RU486). Data are expressed as mean ± SD. *P < 0.05, **P < 0.01 vs. Control group; ^#^*P* < 0.05, ^##^*P* < 0.01 vs. Virus group; ^*&*^*P* < 0.05 vs. “Stress+Virus” group; ^*§§*^*P* < 0^.^01 vs. “CORT + Virus” group

Under stress, CORT is released into the blood, due to the activation of HPA axis.^19^ We therefore examined plasma CORT concentration in mice at indicated periods post-restraint stress. We found that this stress hormone was significantly elevated from day 0 to day 3 after stress and returned to normal physiological level on day 5 (Fig. 2a). To further explore the influence of stress-induced CORT overflow in virus infection, subcutaneous injections of CORT were administered to mice to mimic the situation of restraint stress. Results indicated that the injection of 1 mg/kg of CORT presented equivalent efficacy as restraint stress (on day 3) with similar plasma level of CORT (Fig. 2b). The mortality of mice injected with exogenous CORT (1 mg/kg) was recorded. We found that CORT treatment significantly decreased the survival rate of mice infected with virus (Fig. 2c), which is similar to those from restraint stress mouse model. qRT-PCR (Fig. 2d) and immunohistochemical (IHC) staining (Fig. 2e) results demonstrated that both restraint stress and CORT remarkably elevated NP gene and protein expressions in the lungs of virus-infected mice, which was prohibited by GR antagonist RU486.

The effects of CORT on viral susceptibility were further confirmed in vitro using human lung epithelial A549 cells. Cells were treated with CORT (100 µM) for 48 h and exposed to H1N1 virus infection for 12 h. Data obtained from TCID50 (Fig. 2f), qPCR (Fig. 2g), western blotting (Fig. 2h), and immunofluorescence (Supplementary Fig. S1) assays showed that cells treated with CORT and virus exhibited a dramatic increment in virus titration and NP gene and protein expressions comparing with those in virus-exposed cells. Both in vivo and in vitro evidences indicate the positive correlation between CORT and stress-enhanced susceptibility to influenza virus.

### Stress/CORT impeded MAVS antiviral signaling

**Fig. 3:**
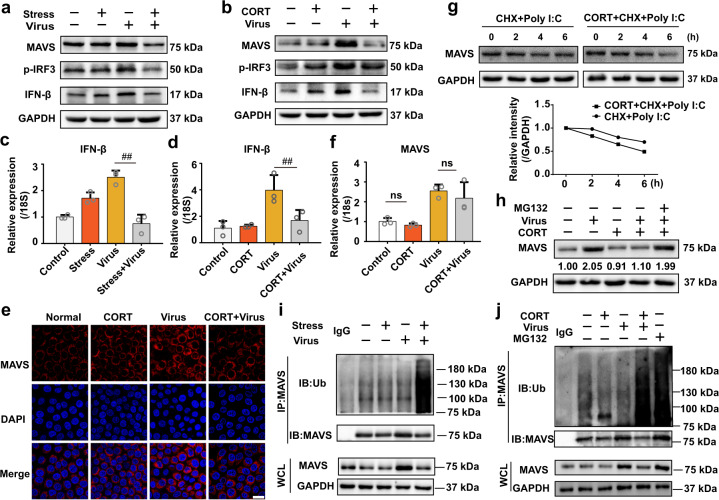
CORT impedes MAVS-transduced IFN-β response triggered by influenza infection. **a**, **b** MAVS, IFN-β, and p-IRF3 protein expressions were determined by western blotting in the lung tissues or A549 cells. **c**, **d** IFN-β mRNA levels were analyzed by RT-qPCR (*n* = 3). **e** A549 cells were pretreated with CORT (100 μM) for 48 h, and then infected with H1N1 virus (10 TCID50). The expression of MAVS was observed by immunostaining at 12 h post infection. Representative immunostaining images of MAVS (red) taken by confocal microscopy. The cell nuclei were stained by DAPI (blue). Scale bars, 20 μm. **f** MAVS gene expression in CORT-loaded A549 cells infected with virus were measured by RT-qPCR at 12 h post infection (*n* = 3). **g** CORT-loaded cells were transfected with poly I:C (50 μM) in the presence of CHX (100 μM) or without CHX, and the protein expression of MAVS was detected by western blotting. **h** CORT-loaded cells were infected with H1N1, and then treated with MG132 (10 μM) for 12 h. MAVS protein expression was determined by western blotting (*n* = 3). **i**, **j** The levels of MAVS ubiquitination were analyzed by western blotting in the lung tissues of mice at 4 d post infection and in A549 cells at 12 h post infection. CORT, corticosterone; CHX, cycloheximide. Data are expressed as mean ± SD. ^*##*^*P* < 0.01 vs. Virus group. ns represents no significance

MAVS can potently activate IRF3 and propagate IFNs and ISGs, which plays a crucial role in defending RNA virus infection.^20,21^ We thus investigated the effect of stress/CORT on MAVS antiviral signaling pathway. Our results showed that H1N1 infection enhanced the protein expression of MAVS (Fig. 3a), and induced the phosphorylation of IRF3 (Fig. 3a) and the expression of IFN-β in the lung tissues of mice (protein in Fig. 3a and gene in Fig. 3c). However, MAVS/IFN-β signaling by virus infection was inhibited by stress/CORT, evidenced by decreased protein level of MAVS, p-IRF3, and IFN-β (Fig. 3a), as well as declined gene level of IFN-β (Fig. 3c). Furthermore, we investigated the impact of CORT on MAVS antiviral signaling in H1N1-infected A549 cells. We firstly determined the change of MAVS level at different time points, and results showed that it increased at 9 and 12 h, and then decreased 24, 36, 48 h post H1N1 infection or RNA viral replication-generated double-stranded RNA mimic poly (I:C) stimulation (Supplementary Fig. S2). An increasing time point (12 h) was chosen to evaluate the influence of CORT. Consistent with in vivo results, MAVS antiviral signaling was also impeded in H1N1/poly (I:C)-treated A549 cells (Fig. 3b, d, and Supplementary Fig. S3). Immunofluorescence staining further verified the effect of CORT on decreasing MAVS protein level (Fig. 3e).

### Stress/CORT initiates ubiquitin-mediated degradation of MAVS

Considering the declined MAVS protein level by stress/CORT, we then determined its gene expression by qRT-PCR. Surprisingly, CORT treatment could not affect MAVS from gene level in virus-infected A549 cells (Fig. 3f). By using protein synthesis inhibitor cycloheximide (CHX), we identified that CORT accelerated MAVS degradation in A549 cells treated with poly (I:C) (Fig. 3g). In the meantime, the treatment of MG132, a proteasome inhibitor, rescued CORT-caused decrease of MAVS in virus-exposed A549 cells (Fig. 3h). The above results prompted us to speculate whether restraint/CORT initiate proteasome-dependent degradation of MAVS. To test this hypothesis, MAVS was immunoprecipitated to measure its ubiquitination level in the lung tissues of mice in response to virus infection with or without stress in vivo and in vitro. Results revealed that restraint and CORT dramatically enhanced the polyubiquitination level of MAVS, in mice (Fig. 3i) and in cells (Fig. 3j) infected with influenza virus. Subsequently, we utilized anti-K48-linked ubiquitin antibody to detect K48-linked ubiquitination of MAVS in vitro and in vivo. Data in Supplementary Fig. S4a and Fig. S4b respectively illustrated that both stress and CORT increased K48-linked polyubiquitination level of MAVS in virus-infected mice or A549 cells. Collectively, the proteasome-dependent degradation of MAVS was facilitated by stress/CORT, thereby prohibiting IFN-β antiviral signaling and increasing influenza virus susceptibility.

Apart from MAVS signaling, the IFN-β response during pathogen infection was also reported to be triggered by nuclear factor kappa B (NF-κB).^22^ In our study, CORT-treated A549 cells with virus infection displayed an obvious increase of NF-κB/p65 compared to cells exposed to virus alone (Supplementary Fig. S6). In the meantime, we discovered that BAY 11-7082, a selective NF-κB inhibitor, significantly downregulated the protein expression of IL-6 and TNF-α, two known NF-κB-regulated inflammatory factors, in CORT-treated and virus-exposed cells (Supplementary Fig. S6). However, BAY 11-7082 only exerted marginal effects on IFN-β protein expression in CORT-treated and virus-infected A549 cells (Supplementary Fig. S6). These results excluded the contribution of NF-κB in CORT-inhibited IFN-β response.

### Stress/CORT-induced viral susceptibility is impaired in MAVS^−/−^ mice

**Fig. 4:**
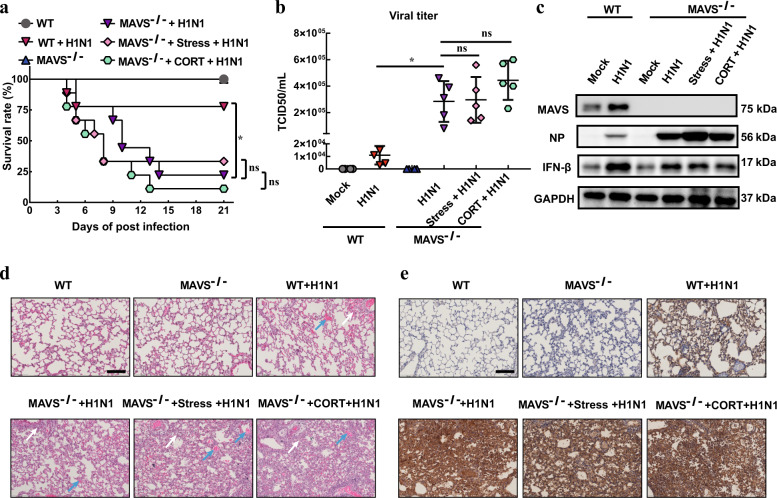
Stress/CORT-induced viral susceptibility is impaired in MAVS^−/−^ mice. WT and MAVS^−/−^ mice were subjected to restraint stress or CORT treatment. After a 2-d recovery, mice was then administrated nasally with H1N1 virus (2×LD50). CORT (1 mg/kg) dissolved in PEG-400 was administered subcutaneously to mice for 2 d before virus infection. **a** The survival rate in each group was monitored for 21 d (*n* = 9). **b** Viral titers were determined by TCID_50_ assay at 4 d post infection (*n* = 4). **c** MAVS/IFN-β/NP protein expressions were analyzed by western blotting at 4 d post infection. **d**, **e** On the 4th day after H1N1 infection, the histological changes and NP protein expression were analyzed by HE and immunohistochemical staining, respectively (*n* = 3). White arrows indicate the presence of inflammatory infiltrates. Blue arrow indicates hemorrhage exudate. Data are expressed as mean ± SD. *P < 0.05 vs. “WT+H1N1” group; ns, *P* > 0.05 vs. “MAVS^−/−^+H1N1” group

MAVS knockout mice were then employed to confirm the role of MAVS antiviral signaling in CORT/stress-evoked virus susceptibility. Results showed that the loss of MAVS resulted in an increased mortality of mice infected with H1N1 (Fig. 4a). Nevertheless, both stress and CORT treatment exerted no significant impact on the survival rate of MAVS^−/−^ mice infected with virus (Fig. 4a). Furthermore, we found that there was no significant difference in viral titers (Fig. 4b), IFN-β protein expression (Fig. 4c), histological changes (Fig. 4d) and NP immunostaining (Fig. 4e) between stress/CORT and non-stress/non-CORT group. All these data obtained in MAVS^−/−^ mice are in sharp contrast with the observations in WT mice, which collectively implies that MAVS antivirus signaling plays an essential role in stress/CORT-induced H1N1 susceptibility.

### Mfn2 is involved in CORT-provoked impediment of MAVS antivirus signaling

**Fig. 5:**
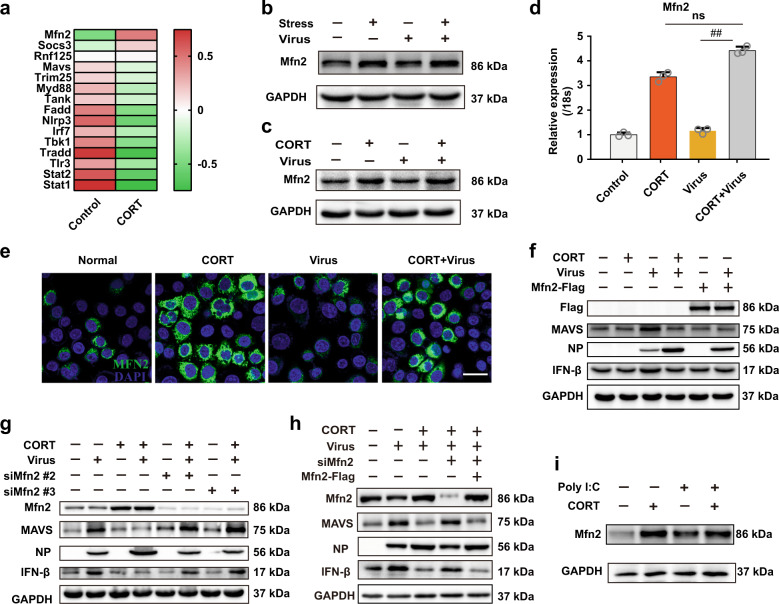
Mfn2 is involved in CORT-prohibited MAVS antivirus signaling. **a** CORT-loaded A549 cells were infected with H1N1, and then sampled for RNA-sequencing analysis at 12 h post infection. Heatmap of RNA-sequencing data displays the mean expressions of antiviral innate immunity-related genes in control and CORT-treated cells (*n* = 3). Colors represent fold-change levels above (red) or below (green) the values in control. **b**, **c** Mfn2 protein expressions were determined by western blotting in the lung tissues of mice after 4 d post infection and in A549 cells at 12 h post infection. **d** Mfn2 gene expression was analyzed by RT-qPCR in CORT-loaded A549 cells at 12 h post infection (*n* = 3). **e** Representative immunostaining images of Mfn2 (green) in A549 cells were taken by confocal microscopy. The cell nuclei were stained by DAPI (blue). Scale bars, 20 μm. **f**–**h** MAVS/IFN-β/NP protein expressions in Mfn2-overexpressed or Mfn2-knockdown A549 cells were analyzed by western blotting at 12 h post infection. **i** A549 cells were treated with or without CORT and poly I:C for 12 h, and the expression of Mfn2 protein was determined by western blotting. CORT, corticosterone. Data are expressed as mean ± SD. ^*##*^*P* < 0.01 vs. Virus group. ns represents no significance

We next investigated the potential mechanism of controlling MAVS degradation. RNA-sequencing analysis of lung tissues from mice was utilized to measure the changes of innate immunity-related genes. Mfn2 was screened out in view of its considerable alteration under CORT treatment (Fig. 5a). In this experiment, we also observed an unaffected mRNA level of MAVS, which was consistent with previous data from qPCR. Furthermore, the effects of stress/CORT on Mfn2 gene and protein levels were measured by qPCR and western blotting. Results indicated that stress and CORT up-regulated both mRNA and protein levels of Mfn2 in both the lung tissues of mice (Fig. 5b) and A549 cells (protein in Fig. 5c and gene in Fig. 5d) in the presence or absence of H1N1 infection. Similar results were observed in poly(I:C)-transfected cells (Fig. 5i). It is worthy to note that virus infection alone had little effect on Mfn2 expression both in vivo and in vitro, suggesting CORT as the sole factor responsible for the alteration of Mfn2.

Next, we knocked down or overexpressed Mfn2 in A549 cells to investigate the role of Mfn2 in MAVS degradation. Similar to CORT treatment, Mfn2 overexpression in cells decreased both MAVS and IFN-β protein levels and promoted viral replication in H1N1-treated cells (Fig. 5f). In contrast, the depletion of Mfn2 by siRNA rescued MAVS antivirus signaling and thus inhibited viral replication (Supplementary Fig. S7a and Fig. 5g), which was rescued by Mfn2 overexpression (Fig. 5h). The above data addressed the importance of Mfn2 in CORT-mediated suppression of MAVS antivirus signaling.

**Fig. 6:**
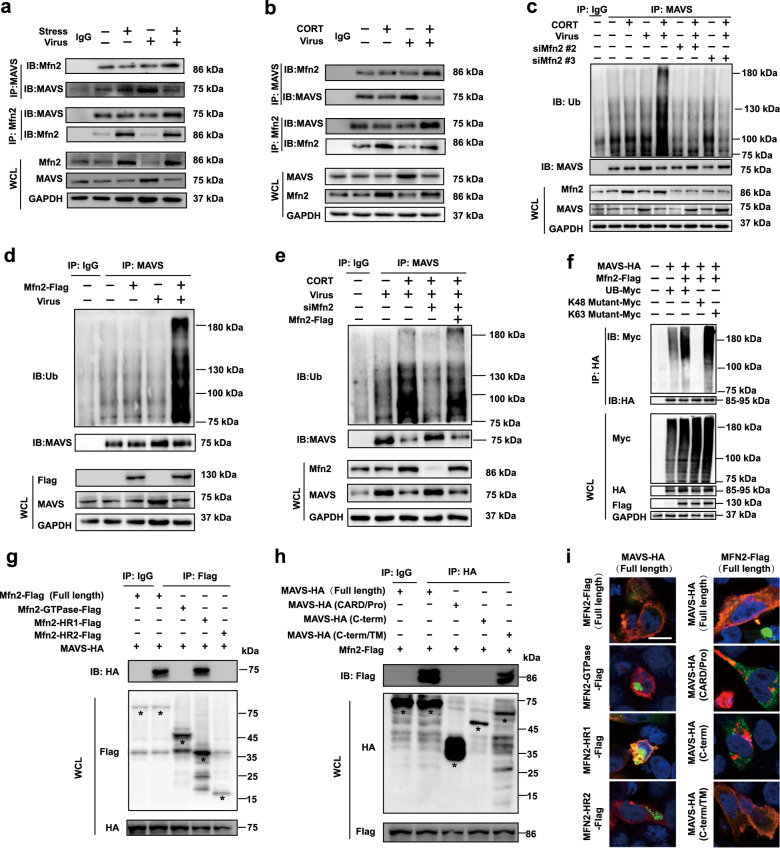
The interaction between Mfn2 and MAVS triggers the ubiquitination of MAVS. **a**, **b** Co-immunoprecipitation assays of MAVS and Mfn2 in the lung tissues of mice at 4 d post infection and in A549 cells at 12 h post infection. Whole lysates were subjected to immunoprecipitation with MAVS or Mfn2 antibody attached to sepharose. Whole-cell lysates (WCL) and immunoprecipitates were analyzed by immunoblotting with an anti-Mfn2 or MAVS antibody. **c**–**e** The levels of MAVS ubiquitination in Mfn2-overexpressed or Mfn2-knockdown A549 cells were measured by western blotting at 12 h post infection. **f** Ubiquitination levels were measured in HEK293T cells co-transfected with pCDNA3.1-MAVS-HA, pCDNA3.1-Mfn2-Flag, pCMV-UB-Myc, pCMV-Ub(K48R)-myc, or pCMV-Ub(K63R)-myc and infected with H1N1. Whole lysates from HEK293T cells were subjected to immunoprecipitation with anti-Myc antibodies attached to sepharose. WCL and immunoprecipitates were analyzed by immunoblotting with anti-HA, anti-Myc, and anti-Flag antibodies. **g**, **h** The interaction between full length and truncations of Mfn2 with MAVS, between full length and truncations of MAVS with Mfn2 was analyzed by co-immunoprecipitation assay in virus-infected HEK293T cells. **i** The co-localization of full length and truncations of MAVS and Mfn2 in virus-infected HEK293T cells was observed under confocal microscopy. CORT, corticosterone

The interaction between Mfn2 and MAVS ignites the ubiquitination of MAVS. Yasukawa et al. have reported that Mfn2 interacted with MAVS to modulate antiviral immunity.^23^ The present study has depicted that CORT accelerated MAVS degradation, and Mfn2 was of great importance in this process. We therefore hypothesized that the interaction between Mfn2 and MAVS contributed to CORT-caused degradation of MAVS. To test this hypothesis, we measured the interaction between Mfn2 and MAVS in stressed mice and CORT-treated A549 cells in the presence or absence of virus. Results showed that Mfn2 was bound to MAVS both in vivo and in vitro, while stress/CORT enhanced their interaction (Fig. 6a, b). Confocal images also indicated that CORT promoted the co-localization of MAVS and Mfn2 in virus-infected A549 cells (Supplementary Fig. S5).

Next, we explored the impact of Mfn2 on MAVS ubiquitination. Results showed that Mfn2 overexpression increased the polyubiquitination level of MAVS in A549 cells infected with virus (Fig. 6d). By contrast, the depletion of Mfn2 by siRNA decreased the ubiquitination level (Fig. 6c), which was recovered by Mfn2 overexpression (Fig. 6e). We further transfected with plasmids encoding MAVS-HA, Mfn2-Flag, and Ub-Myc in virus-infected 293T cells. The overexpression of Mfn2-flag significantly increased the polyubiquitination of MAVS (Fig. 6f). When we examined the linkage of MAVS ubiquitination, we found that Mfn2 regulated only K48, but not K63 ubiquitin chains of MAVS (Fig. 6f).

The interacting regions between Mfn2 and MAVS were then explored. Different truncated Mfn2 and MAVS were overexpressed in HEK293T cells followed by virus infection. Using a Co-IP approach, 4,3 hydrophobic heptad repeat (HR1) region in Mfn2 (amino acid residues 369–598) was revealed as MAVS-interacting region (Fig. 6g). Results yielded from truncated MAVS suggested that the C-term/TM region of MAVS (amino acid residues 201–540) was required for the interaction with Mfn2 (Fig. 6h). This observation was further confirmed by the co-localization of Mfn2-HR1 and MAVS-C-term/TM Mfn2 observed in immunostaining images in virus-infected HEK293T cells (Fig. 6i).

### Identification of SYVN1 as the E3 ligase in Mfn2-mediated MAVS ubiquitination

**Fig. 7:**
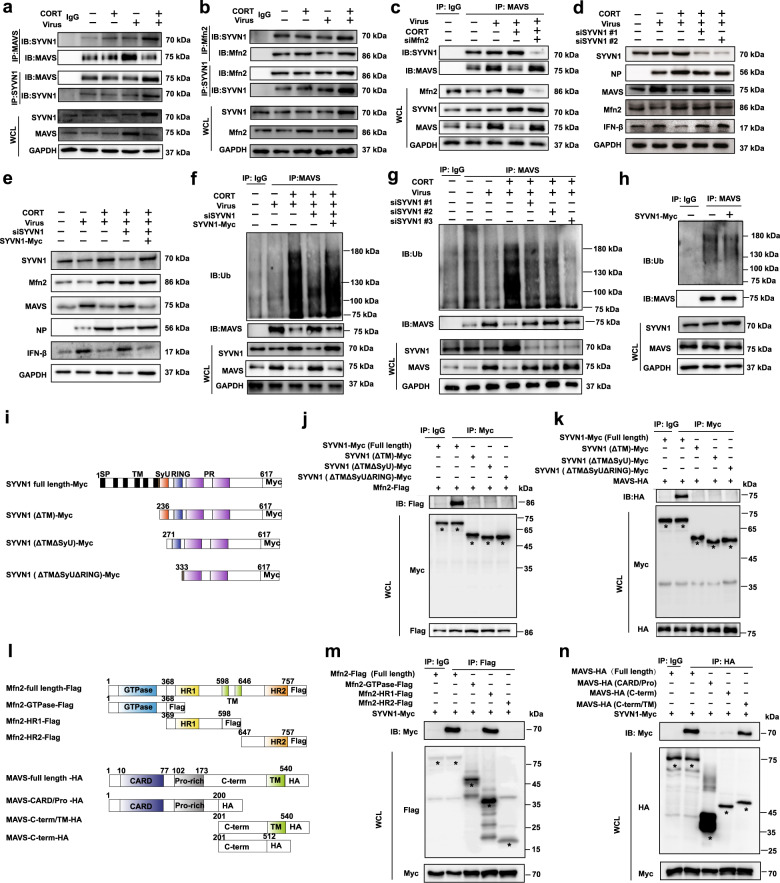
Identification of SYVN1 as the E3 ligase in Mfn2-mediated MAVS ubiquitination. **a** Co-immunoprecipitation assay of MAVS and SYVN1 in A549 cells. At 12 h post infection, WCL were subjected to immunoprecipitation with MAVS or SYVN1 antibody attached to sepharose. WCL and the immunoprecipitates were analyzed by immunoblotting with an anti-SYVN1 or MAVS antibody. **b** Co-immunoprecipitation assay of Mfn2 and SYVN1 in A549 cells. WCL were subjected to immunoprecipitation with Mfn2 or SYVN1 antibody attached to sepharose. WCL and immunoprecipitates were analyzed by immunoblotting with an anti-SYVN1 or Mfn2 antibody. **c** The interaction of MAVS and SYVN1 in Mfn2-knockdown A549 cells were analyzed by co-immunoprecipitation assay at 12h post infection. **d**, **e** NP/MAVS/IFN-β protein expressions in SYVN1-knockdown or overexpressed A549 cells were measured by western blotting at 12 h post infection. **f**–**h** MAVS ubiquitination levels in SYVN1-knockdown and overexpressed A549 cells were analyzed by western blotting at 12 h post infection. **i** The truncated variants of Myc-tagged SYVN1 are depicted. **j**, **k** The interaction of truncated Myc-tagged SYVN1 variants with Flag-tagged Mfn2 or HA-tagged MAVS in virus-infected HEK293T cells were analyzed by co-immunoprecipitation assays. **l** The truncated variants of Flag-tagged Mfn2 or HA-tagged MAVS are depicted. **m**, **n** The interaction of Myc-tagged SYVN1 with Flag-tagged Mfn2 depletion mutants or HA-tagged MAVS depletion mutants in virus-infected HEK293T cells were analyzed by co-immunoprecipitation assays. CORT, corticosterone

E3 ligase plays an indispensable role in the ubiquitination process.^24^ In order to define the E3 ligase in Mfn2-mediated MAVS ubiquitination during influenza infection, we initially resorted to UbiBrowser database to make a prediction,^25^ and SYVN1 was estimated as a potential E3 ligase with the highest score. To verify this prediction, co-immunoprecipitation (Co-IP) was performed in A549 cells with virus infection. It was shown that CORT enhanced the interaction between MAVS and SYVN1(Fig. 7a) and the interaction of SYVN1 and Mfn2, respectively (Fig. 7b). When Mfn2 was knocked down by siRNA, CORT-caused interaction between MAVS and SYVN1 was diminished (Fig. 7c). Collectively, the interaction between any two of the three proteins, namely Mfn2, MAVS, and SYVN1, was delineated in stress/CORT- plus virus- treated mice or cells. This intriguing finding prompted us to further determine whether SYVN1 was of significant importance in MAVS ubiquitination and MAVS/IFN-β signaling. In this regard, we found that the loss of SYVN1 by siRNA decreased NP expression, but increased MAVS and IFN-β expressions (Supplementary Fig. S7 and Fig. 7d). Curiously, the protein expression of Mfn2 was not affected when SYVN1 was knock-downed (Fig. 7d, e), excluding the possibility of SYVN1 as an E3 ligase of Mfn2. More importantly, the depletion of SYVN1 dramatically decreased the ubiquitination level of MAVS (Fig. 7g). Further loss- and gain-function experiments showed that SYVN1 overexpression recovered siSYVN1-caused changes of protein and ubiquitination levels of MAVS in CORT- plus virus-treated cells (Fig. 7e, f). Nevertheless, in the absence of virus and CORT, single SYVN1 overexpression did not engender obvious impact on MAVS ubiquitination (Fig. 7h), indirectly suggesting the importance of Mfn2 as a scaffolding protein to bring MAVS and SYVN1 together.

Next, MAVS or Mfn2 binding region with SYVN1 was investigated in virus-infected HEK293T cells, respectively. Cells were co-transfected with full length or deletion mutants of Myc-tagged SYVN1 and Flag-tagged Mfn2 or HA-tagged MAVS plasmids. After a 24-h transfection, cells were infected with H1N1 for 12 h. Cells were harvested to conduct co-immunoprecipitation experiments. The protein complexes were precipitated with anti‑Myc antibodies for truncated SYVN1 variants, and were detected using anti-Flag or anti‑HA antibodies. As a result, all SYVN1 truncated variants lacking the transmembrane domain (SYVN1 ΔTM) could not interact with Mfn2 or MAVS (Fig. 7i–k), indicating the pivotal role of TM domain in SYVN1 in interacting with both Mfn2 and MAVS. Furthermore, to identify the region of Mfn2 or MAVS that interact with SYVN1, an analogous approach with deletion mutants of Mfn2 or MAVS were co-transfected with full length of SYVN1 in virus-infected HEK293T cells. We found that HR1 region of Mfn2 was required for its interaction with SYVN1 (Fig. 7i, m). Meanwhile, C-term/TM region of MAVS, which is required for the interaction with Mfn2, was also necessary for the interaction with SYVN1 (Fig. 7i, n).

Collectively, these data suggested that the increment of Mfn2 caused by CORT recruited E3 ligase SYVN1 to initiate the ubiquitination of MAVS, thus impeding MAVS/IFN-β response.

## Discussion

Individuals under stress have weakened immunity and most likely need hospitalization after influenza virus infection, raising the possibility that stress is a susceptible factor of influenza infection.^26^ This hypothesis has been supported by increasing evidences,^13,14,27^ in addition to our research. When we attempted to elucidate the mechanism responsible for stress-induced influenza susceptibility, CORT (one type of GCs, an indicator of stress response) was defined as the major culprit. In fact, due to its well-known immunocompromising role, the link between GCs and virus infection has aroused people’s attention for many years. For instance, Dobbs et al. reported that CORT induced by restraint stress suppressed the influenza virus (A/PR/8/34)-specific production of Th1-type (IL-2) and Th2-type (IL-10) cytokines by cells from lymph nodes and spleens to increase influenza mortality.^28^ However, more efforts are required to explore the influence of CORT on RNA virus infection-specific MAVS antivirus signaling, although there have already been interesting reports suggesting that GR can compete with IRF3 for GRIP1 to inhibit IFN-β transcription, and also can restrain the STAT transcription complex activation to inhibit the expression of IFN-β stimulated genes.^29^ In our research, we reported, for the first time to our knowledge, that the production of CORT resulting from HPA axis activation by restraint stress disturbed the protein stability of MAVS via Mfn2-SYVN1 axis to suppress innate immunity.

Before determining the impact of CORT/stress on MAVS antivirus signaling, we firstly examined the change of MAVS amount at different time points post H1N1 infection or poly I:C stimulation in A549 cells. Our results showed that the protein level of MAVS increased at the earlier times (9 and 12 h), and then decreased at the latter times (24, 36, 48 h) post virus infection or poly I:C (Supplementary Fig. S2). This time point experiment demonstrates that H1N1 infection has different effects on MAVS level at different infection stages. How viral infection reduces MAVS level at later time points have been explored by previous reports, from the perspective of miRNA-regulated expression,^30^ ubiquitination/autophagy-mediated degradation.^31,32^ Conversely, there are some studies suggesting that MAVS can be deubiquitinated upon virus infection,^33^ giving us a hint that deubiquitination modification might contribute to the increased MAVS protein level, urgently limiting the replication of virus at the earlier times post infection. As we revealed that both virus infection and poly I:C has similar effect on MAVS, dsRNA other than virus proteins are indicated to be involved in this process. Taken the above findings into consideration, an increasing time point of MAVS level (12 h post virus infection) was chosen to evaluate the influence of CORT.

MAVS is located in the mitochondrial outer membrane, along with several other mitochondrial proteins such as NLRX1 and Mfn2 associated with MAVS, which may either negatively or positively regulate MAVS function and influence the immune balance.^23,34,35^ Consistent with a previous study reported by Yasukawa et al.,^23^ we observed that Mfn2 interacted with MAVS and inhibited MAVS-mediated antiviral responses as well. Specifically, the production of CORT, induced by stress response, significantly increased Mfn2 gene and protein levels in the presence or absence of virus. The enhanced Mfn2 interacted with MAVS to inhibit the phosphorylation of IRF3 and downstream IFN-β transcription. Furthermore, we found that Mfn2 critically affected the ubiquitin degradation of MAVS, as shown in Co-IP and ubiquitination assays in vivo and in vitro. Intriguingly, although stress/CORT exposure can increase the expression of Mfn2, but has no effect on both the protein level and ubiquitination level of MAVS in the absence of virus. In accordance with these results, overexpression of Mfn2 alone also has no-decreasing effect on both the protein level and ubiquitination level of MAVS. By sharp contrast, in the presence of virus, stress/CORT induces ubiquitination degradation of MAVS in a Mfn2-dependent manner. This discrepancy strongly indicated that virus infection warranted CORT-induced degradation of MAVS via Mfn2 upregulation. We speculate that the activation of MAVS by virus is the prerequisite for the interaction of Mfn2 and MAVS and the resultant MAVS ubiquitination.

Another emerging question is how the interaction between Mfn2 and MAVS ignites MAVS ubiquitination. We identified SYVN1 as the E3 ligase of MAVS under stress/CORT exposure. More interestingly, Mfn2 could interact with both MAVS and SYVN1, while the depletion of Mfn2 by siRNA abolished the suppressive effect of CORT on the polyubiquitination level of MAVS and the subsequent IFN-β response. We thus conclude that Mfn2 serves as a scaffolding protein to bring the substrate MAVS and the E3 ligase SYVN1 together, initiating the polyubiquitination-mediated degradation of MAVS. Nevertheless, some questions remain unresolved. For instance, SYVN1 has been reported to mainly locate in endoplasmic reticulum (ER).^36^ Therefore, it remains uncertain how E3 ligase is trans-located from ER to mitochondria. It also remains unknown whether CORT affects other E3 ligases of MAVS, including AIP4,^31^ TRIM25,^37,38^ TRIM31,^39^ Smurf1,^40^ and Smurf2.^41^ The clarification of the roles of these E3 ligases may help us fully address the mechanism of CORT-provoked MAVS ubiquitination.

In sum, our study reveals CORT as an essential regulator of MAVS-dependent IFN-β response, in response to virus infection under stress status. Mechanistically, Mfn2 serves as a scaffolding protein to bring E3 ligase SYVN1 to the substrate MAVS, which switches on the ubiquitin-mediated degradation of MAVS. In the end, through a series of co-immunoprecipitation experiments, we clarified an interaction between any two regions of Mfn2 (HR1), MAVS (C-terminal/TM) and SYVN1 (TM). This study presents a novel insight into the stress-induced susceptibility to influenza virus and provides innovative strategies in treating influenza infection.

## Materials and methods

### Reagents

CORT, RU486, MG132, and CHX were purchased from Sigma (St. Louis, Missouri, USA). Ethyl acetate, ether, and methanol were purchased from Shandong Yuwang Industrial Co. (Shandong, China). Protein A-agarose beads, anti-phospho-IRF3 rabbit monoclonal antibody, anti-TNF-α rabbit polyclone antibody, and anti-IL-6 rabbit monoclonal antibody were obtained from Cell Signaling Technology (Beverly, MA, USA). Anti-MAVS rabbit polyclonal antibody was purchased from Proteintech (Rosemont, Illinois, USA). Anti-mitofusin2 (Mfn2) mouse monoclonal antibody, anti-IFN-β rabbit polyclonal antibody, and anti-Ub rabbit monoclonal antibody were purchased from Abcam (Cambridge, MA, UK). Anti-NP rabbit polyclonal was purchased from Genetex (San Antonio, Texas, USA). Anti-β-actin mouse monoclonal antibody and HRP-labeled secondary antibody were purchased from Hangzhou Fu De Biological Technology Co. (Shanghai, China). Chemicals were obtained from Hangzhou Fu De Biological Technology Co. (Shanghai, China) unless otherwise indicated.

### Virus

Embryonated SPF chicken eggs were provided by Genetimes Technology (Shanghai, China). Influenza virus A/FM/1/47 (H1N1) (strain A/Fort Monmouth/1/1947 H1N1) was donated by Prof. Jian-Xin Chen, College of Veterinary Medicine, South China Agricultural University (Guangzhou, China). Its viral titers were counted by plaque assays.^42^ The virus strain was adapted for lethality in mice after three passages, and propagated in embryonated SPF chicken eggs (Genetimes Technology Ltd., Shanghai, China). The LD_50_ was calculated in mice after serial dilution of the stock. Double amounts of LD_50_ were used in all animal experiments for viral challenge. Mice were anesthetized with ether followed by intranasal injection of influenza A/FM/1/47 (H1N1) virus in 35 μL of PBS, while control mice were infected with an equivalent volume of dilution allantoic fluid.

### Animals and treatments

Male specific pathogen-free (SPF) Kunming mice (13–15 g) were purchased from Guangdong Medical Laboratory Animal Center (Guangzhou, China). MAVS knockout C57BL/6 mice (The Jackson Laboratory, B6.129-Mavs<tm1zjc>/J, Stock No: 008634) were generously provided by Prof. Zhenyou Jiang’s group. All mice were housed in-groups in cages with bedding, controlled temperature (23 ± 2 °C), illumination (12 h light/dark cycles), and humidity (50 ± 5%). Mice were adapted to the facility for 1 week before experiments. All animal experiments were performed in accordance with the National Institutes of Health’s Guide for the Care and Use of Laboratory Animals (NIH publication No. 80-23, revised in 1996) and were approved by the Animal Ethics Committee of Jinan University. The influenza-related pathogenic operation was performed in the Animal Biosafety Level 2 Laboratory.

In the first batch of animal experiments, Kunming mice were randomly divided into three groups (*n* = 10), including control, virus, and “restraint stress+virus”. For restraint stress, mice were physically subjected to restraint in a polypropylene restraint tube with holes for 22 h and recovered for 2 d. Then, animals were anesthetized and an approximate 2×LD_50_ amount of H1N1 virus (35 μL) was instilled into the nares. When mouse showed typical influenza symptoms, including weight loss, hunched back, ruffled fur, altered respiration, and unresponsiveness, as well as decreased body weight (over 1 g/d), it was deemed in morbid state. The morbidity and mortality in each group were examined by recording the percentage of animals remaining healthy or surviving due to H1N1 virus exposure for 21 d or until death. Viral NP gene and protein expression in the mice lungs were determined on the 4th day after H1N1 infection. The schematic protocol is shown in Fig. 1a.

In the second batch of animal experiments, Kunming mice were randomly divided into four groups (*n* = 10), including control, restraint stress, CORT (1 mg/kg), and CORT (2 mg/kg). Mice in restraint stress group were subjected to restraint for 22 h and recovered for 2 d. Mice in CORT groups were subcutaneously injected with 1 or 2 mg/kg CORT (in polyethylene glycol 400, PEG-400) for 2 d. The animals were sacrificed to collect plasma and the CORT level was determined.

In the third batch of animal experiments, Kunming mice were divided into five groups (*n* = 10), including control, virus, “stress+virus”, “CORT + virus”, “stress+ virus+RU486”. CORT (1 mg/kg) dissolved in PEG-400 was administered subcutaneously to mice for 2 d before virus infection. Two hours before each CORT treatment, GR antagonist RU486 (25 mg/kg) was subcutaneously injected into mice. Body weights and survivals in each group were recorded daily for 21 d or until death. Mortality was presented as the percentage of animals surviving to the total number of mice against the H1N1 virus exposure.

In the 4th batch of animal experiments, Kunming mice were grouped and treated as the third experiment. Four day after virus infection, mice were weighed and anesthetized by ethyl ether. Samples of lung tissue were collected for qRT-PCR, western blotting, histopathologic analysis, IHC staining, and Co-IP analysis.

In the 5th batch of animal experiments, MAVS^−/−^ C57BL/6 mice were employed to confirm the role of MAVS antivirus signaling in stress/CORT-induced H1N1 susceptibility. WT and MAVS^−/−^ mice were divided into six groups, including WT, “WT + virus”, “MAVS^−/−^ + virus” “MAVS^−/−^ + stress+virus”, and “MAVS^−/−^ + CORT + virus”. Mortality was examined over a period of 21 d by recording the survival of mice in each group. The protein expressions of MAVS, IFN-β, and NP, and histological changes of lung tissues were examined on the 4th day after H1N1 infection.

### Cell culture and treatment

Human lung adenocarcinoma epithelial A549 cells, HEK293T cells, and MDCK cells were cultured in Dulbecco’s modified Eagle medium (DMEM; Invitrogen, Waltham, MA, USA) and supplemented with 10% fetal bovine serum (FBS) at 37 °C in 5% CO_2_ until 80–85% of confluence. After a 48-h incubation with CORT, cells were infected with 10 TCID_50_ H1N1 virus 2 h for adsorption, and then washed and cultured for another 12 h.

### Plasmids/siRNA transfection

The small interfering RNA (siRNA), siRNA targeting Mfn2 and SYVN1 (RiboBio Co., Ltd., Guangzhou, China) were transfected to A549 cells, and a non-targeting siRNA was used as negative control. The transfection was conducted using lipofectamine 2000 (Invitrogen, Waltham, MA, USA), as described in the manufacturer’s instructions. Six hours post transfection, cells were treated with CORT (100 µM) for 48 h. Then cells were infected with 10 TCID_50_ H1N1 virus for 2 h adsorption, washed, cultured for another 12 h, and collected for analysis.

Plasmids including pcDNA3.1-MAVS-HA, pcDNA3.1-MAVS(C-Term)-HA, pcDNA3.1-MAVS(C-Term/TM)-HA, pcDNA3.1-MAVS (CARD/Pro)-HA, pcDNA3.1-Mfn2-Flag, pcDNA3.1-Mfn2(GTPase)-Flag, pcDNA3.1-Mfn2(HR1)-Flag, pcDNA3.1-Mfn2(HR2)-Flag, pcDNA3.1-SYVN1-myc, pcDNA3.1-SYVN1-myc, pcDNA3.1-SYVN1(∆TM)-myc, pcDNA3.1-SYVN1(∆TM∆SyU)-myc, pcDNA3.1-SYVN1(∆TM∆SyU∆RING)-myc, pCMV-Ub-myc, pCMV-Ub(K48R)-myc, and pCMV-Ub(K63R)-myc were transfected to A549 or HEK293T cells using lipofectamine 2000. After 6 h, cells were treated with CORT for 48 h and then infected with 10 TCID_50_ H1N1 virus as described above.

### Histopathologic analysis

Lung samples were fixed in 4% paraformaldehyde and embedded in paraffin. Lung sections were sliced (4 μm) and mounted on microscopic slides. At least 5–10 fields of 3 sections per animal and 4 animals per experimental group were evaluated. Lung histopathologic changes in influenza virus-infected animals were examined by H&E staining under a light microscope (Olympus, Tokyo, Japan).

### IHC staining

IHC staining was used to evaluate the viral NP labeling in the lung section. Slides were cut longitudinally, dewaxed in xylene (twice for 5 min), rehydrated in 100% ethanol (twice for 2 min), 95% ethanol (for 1 min), and 80% ethanol (for 1 min). Antigen retrieval was performed by boiling slides for 30 min in 1 mM EDTA buffer (pH 8.0). At room temperature, slides were blocked in 1% FBS, 0.4% TritonX-100 for 1 h, and then incubated with rabbit anti-NP antibody overnight. Sections were washed twice with blocking buffer and subsequently incubated with goat anti-mouse secondary antibody. The sections were then stained with hematoxylin for 2 min before being examined by microscopy.

### Determination of CORT levels in plasma

Blood was centrifuged at 4500 *×* *g* for 10 min to collect the plasma. CORT was extracted from the plasma and quantified by HPLC.^13^ Plasma (0.5 mL) was mixed with 30 µL of cortisone solution (0.125 mg/mL methanol-water 60:40 v/v) as an internal standard. Steroids were extracted by adding 2 mL of ethyl acetate and mixed thoroughly. The column (5C18, 4.6 × 100 mm; particle size 5 µm; Waters Corp., Milford, Massachusetts, USA) was equilibrated using HPLC grade acetonitrile-water (38:72, v/v) with a flow rate of 1 mL/min.

### RT-qPCR

According to the manufacturer’s protocol, total RNA was extracted from mice lung tissues or A549 cells using TRIzol reagent (Invitrogen, Carlsbad, CA). RNA concentrations were determined by optical density measurement at 260 nm on a spectrophotometer (Thermo, Waltham, MA, USA), and cDNA was synthesized from the purified RNA by both random and oligo (dT) priming using an iScript cDNA synthesis kit (TransGen Biotech, Beijing, China). Intracellular NP and IFN-β RNA levels were measured using the SYBR green method (Applied Biosystems) on a reverse transcription (RT) machine (CFX Connect^TM^; Applied Biosystems) and the relative quantitation method. The samples were normalized by subtracting the threshold cycle values of 18S. The fold induction of viral RNA or innate immune genes over the levels of induction for either mock-infected cells or DMSO-treated control cells was calculated. The specific primers were designed using Primer Premier 5.0 based on the genome sequences of Influenza A virus NP, and mouse or human 18S, MAVS, IFN-β, and Mfn2. Their primer sequences were as follows: H1N1 NP (Forward, 5′-CAGGTACTGGGCCATAAGGAC-3′; Reverse, 5′-GCATTGTCTCCGAAGAAATAAG-3′), human IFNB1 (Forward, 5′-CCAACAAGTGTCTCCTCCAA-3′; Reverse, 5′-ATAGTCTCATTCCAGCCA-GT-3′), human Mfn2 (Forward, 5′-GCAATGTCCCTGCTCTTCTCTC-3′; Reverse, 5′-TCTTGGCACTCCTCTTTTCCTCT-3′), human MAVS (Forward, 5′-CTTCCCCTGTGTTCACCTTTCTG-3′; Reverse, 5′-CATTGTCCCCTGGG-TCCTTCA-3′), human 18S (Forward, 5′-CCAGTAAGTGCGGGTCATAAGC-3′; Reverse, 5′-GCCTCACTAAACCATCCAATCGG -3′), mouse IFNB1 (Forward, 5′-TCGGACCACCATCCAGGCGT-3′; Reverse, 5′-ACCTCACCTACAGGG-CGGACT-3′), Mouse 18S (Forward: 5′-CGGACAGGATTGACAGATTGATAGC-3′; Reverse, 5′-TGCCAGAGTCTCGTTCGTTATCG-3′).

### Western blotting

The protein from lung tissues and A549 and HEK293T cells was determined by pierce BAC protein assay kit (Thermo Scientific, USA). Typical 30 µg proteins were loaded on 10 or 15% SDS-PAGE gel. Electrophoresed protein was transferred to polyvinylidene fluoride membrane (Millipore, Massachusetts, USA), probed with antibodies against MAVS, Mfn2, NF-κB p65, NP, p-IRF3, IFN-β, TNF-α, or IL-6, respectively, and followed by horseradish peroxidase (HPR) conjugated secondary antibodies (anti-rabbit and anti-mouse IgG, Santa Cruz, CA, USA). The bound secondary antibody was detected using pierce ECL western blotting substrate (Thermo Fisher Scientific, Santa Fe, NM, USA).

### Co-IP and immunoblot analysis

To Co-IP endogenous proteins, lung tissues or A549 cell samples were harvested and lysed with RIPA buffer supplemented with protease inhibitor cocktails and phosphatase inhibitor cocktails. Antibodies were recovered by incubating with recombinant protein A agarose for 2 h, followed by overnight incubation with anti-MAVS, anti-Mfn2, anti-SYVN1, anti-Myc, anti-Flag, or anti-HA antibody at 4 °C. The protein A agarose was collected and washed four times with lysis buffer after immunoprecipitation. The immunoprecipitates were separated by SDS-PAGE and analyzed by western blotting.

### RNA sequencing

RNA sequencing was performed by RiboBio Co., Ltd. (Guangzhou, China) with the Illumina HiSeq 2500. RNA-seq data was aligned to the Ensembl v73 transcript annotations using bowtie and RSEM as previously described.^43^ All other bioinformatic analysis was performed using glbase.^44^

### Immunofluorescence assay

Cells from each group were fixed with 4% paraformaldehyde for 30 min and then permeabilized with 0.1% Triton-X100 for 5 min. After blocking with 2% bovine serum albumin for 20 min and staining with NP, the cells were placed in a rabbit polyclonal MAVS or Mfn2 in a dilution of 1:100 at 4 °C overnight. The secondary antibodies conjugated with Alexa Fluor 488 anti-mouse IgG in a dilution of 1:200 and 555 conjugated goat-rabbit IgG in a dilution of 1:200 were applied for 50 min at room temperature. Nuclei were counterstained with DAPI, and cells were visualized and analyzed using a confocal laser-scanning microscopy.

### Data analysis

All data were expressed as means ± SD. The statistical differences between the groups were assessed (SPSS, Version 15, USA) using one-way analysis of variance (ANOVA) for multiple comparisons with Tukey post hoc test to determine statistical significance. Survival curves were estimated using Kaplan–Meier method and log-rank test was used to assess the group differences using GraphPad Prism5 (GraphPad Software, La Jolla, CA). Differences were considered significant at values of *P* < 0.05. All statistical tests were two-sided.

## Supplementary information

Supplemental Material

Supplementary Fig. S1

Supplementary Fig. S2

Supplementary Fig. S3

Supplementary Fig. S4

Supplementary Fig. S5

Supplementary Fig. S6

Supplementary Fig. S7

## Data Availability

All data and materials are available to the researchers once published.
