# Supplementary material for: Novel insights into stress-induced susceptibility to influenza: corticosterone impacts interferon-β responses by Mfn2-mediated ubiquitin degradation of MAVS
Source: Signal Transduct Target Ther. 2020 Sep 18;5:202. doi: 10.1038/s41392-020-00238-z (PMC7499204; doi:10.1038/s41392-020-00238-z)
Supplement: Supplementary file 1 — Supplemental Material [file 41392_2020_238_MOESM1_ESM.pdf]

Supplemental Material

Novel insights into stress-induced susceptibility to influenza: corticosterone impacts interferon-β responses by Mfn2-mediated ubiquitin degradation of MAVS

Zhuo Luo[1,2,3#], Li-Fang Liu[1,2,3#], Ying-Nan Jiang[4#], Lu-Ping Tang[1,2,3], Wen Li[1,2,3], Shu-Hua Ouyang[1,2,3], Long-Fang Tu[1,2,3], Yan-Ping Wu[1,2,3], Hai-Biao Gong[1,2,3], Chang-Yu Yan[1,2,3], Shan Jiang[1,2,3], Yu-Hui Lu[1,2,3], Tongzheng Liu[5], Zhenyou Jiang[6], Hiroshi Kurihara[1,2,3], Yang Yu[2,3], Xin-Sheng Yao[2,3,4*], Yi-Fang Li[1,2,3*], Rong-Rong He[1,2,3*]

[1]Guangdong Engineering Research Center of Chinese Medicine & Disease Susceptibility, Jinan University, Guangzhou 510632, China; [2]International Cooperative Laboratory of Traditional Chinese Medicine Modernization and Innovative Drug Development of Chinese Ministry of Education (MOE), College of Pharmacy, Jinan University, Guangzhou 510632, China; [3]Guangdong Province Key Laboratory of Pharmacodynamic Constituents of TCM and New Drugs Research， College of Pharmacy, Jinan University, Guangzhou 510632, China; [4]School of Traditional Chinese Materia Medica, Shenyang Pharmaceutical University , Shenyang, 110016, China; [5]Institute of Tumor Pharmacology, College of Pharmacy, Jinan University, Guangzhou 510632, China; [6]Department of Microbiology and Immunology, Basic Medicine College, Jinan University, GuangZhou 510632, China

*Correspondence: Rong-Rong He (rongronghe@jnu.edu.cn) or Yi-Fang Li (liyifang706@jnu.edu.cn), Xin-Sheng Yao (tyaoxs@jnu.edu.cn)

[#]These authors contributed equally: Zhuo Luo, Li-Fang Liu and Ying-Nan Jiang

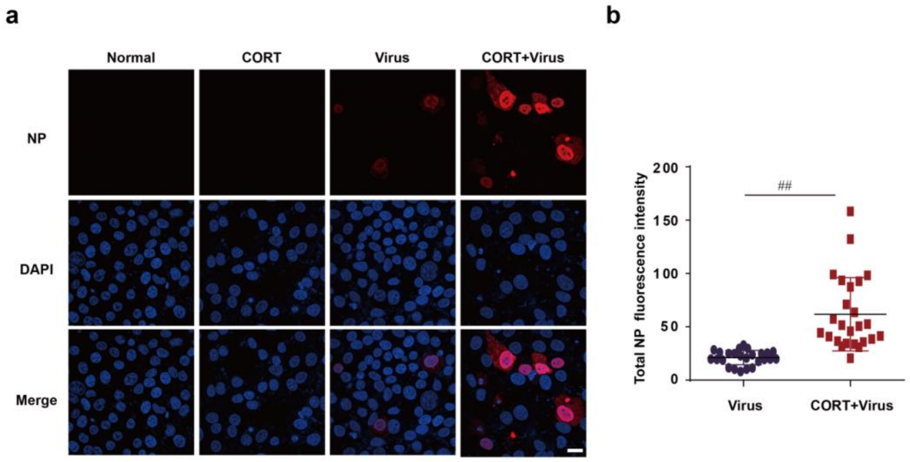

Supplementary Fig. S1. CORT enhances susceptibility to influenza virus *in vitro*.
a A549 cells were treated with CORT for 48 h and infected with 10 $TCID_{50}$ of virus for 12 h. NP protein level were detected by immunostaining. Scale bars, 20 μm. **b** the fluorescence intensity of NP protein in infected cell were quantified by image J. CORT, corticosterone; Data are expressed as mean ± SD. $^{\#\#}p < 0.01$, vs. Virus group.

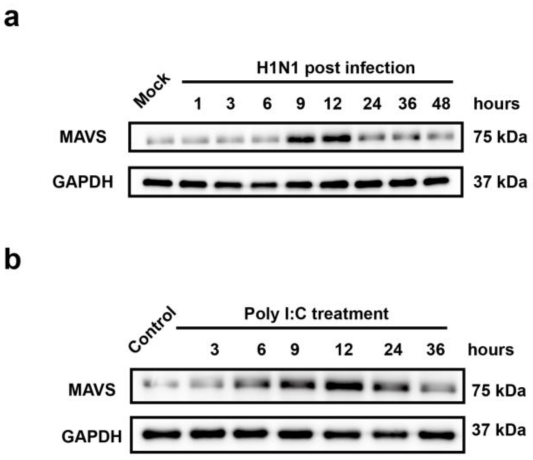

Supplementary Fig. S2. Effect of H1N1 or poly(I:C) on MAVS protein expression at different time points in A549 cells. **a** Cells were infected with H1N1 virus (10 $TCID_{50}$) for 2 h, and the expression of MAVS protein were determined by western blotting at the indicated time point. **b** Cells were transfected with 50 ng/ml poly(I:C) for different periods, and MAVS protein level were detected by western blotting.

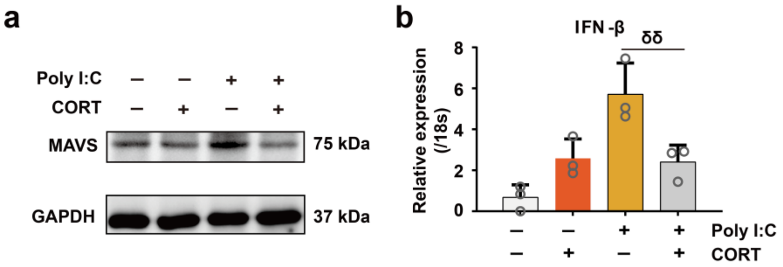

Supplementary Fig. S3. CORT hinders MAVS-transduced IFN-β response in Poly I:C-transfected A549 cells. A549 cells were pre-treated with CORT (100 μM) for 48 h, and then stimulated by Poly I:C for 12 h. **a, b** Cells were collected to determine the expression of MAVS protein and IFN-β gene by western blotting and RT-qPCR, respectively (n=3). CORT, corticosterone. Data are expressed as mean ± SD. $^{\delta\delta}p < 0.01$ vs. "Poly I:C" group.

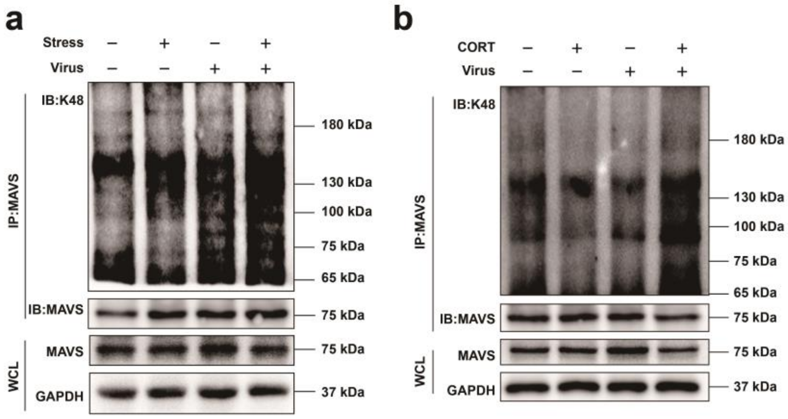

Supplementary Fig. S4. Stress/CORT increases K48-linked polyubiquitination level of MAVS in virus-infected mice or A549 cells. After restraint stress or CORT treatment, mice and A549 cells were challenged with H1N1 virus. **a, b** The K48-linked polyubiquitination level of MAVS in the lung tissues of mice (**a**) and cells (**b**) were analyzed by western blotting. CORT, corticosterone.

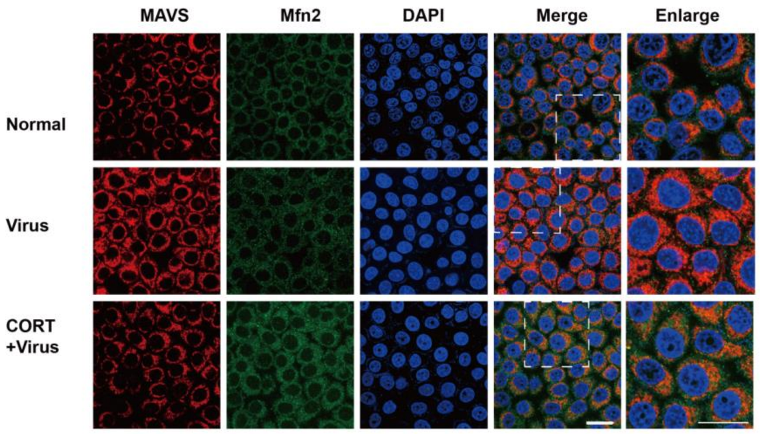

Supplementary Fig. S5. The co-localization of Mfn2 and MAVS in A549 cells. Cells were pretreated with CORT (100 μM) for 48 h, and then infected with 10 TCID50 H1N1 virus. At 12 post infection, Cells were visualized with Mfn2 (green) and MAVS (red) by confocal microscopy. The cell nuclei were stained by DAPI. Scale bars, 20 μm.

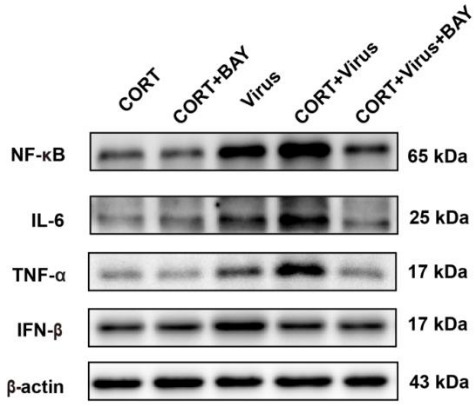

Supplementary Fig. S6. Inhibition of NF-κB activation has little influence on IFN-β protein expression. A549 cells were treated with 5 μM BAY11-7082 and 100 μM CORT for 48 h, and then infected with 10TCID50 virus. Cells were collected to perform western blotting. CORT, corticosterone. BAY, BAY 11-7082 (a selective NF-κB inhibitor).

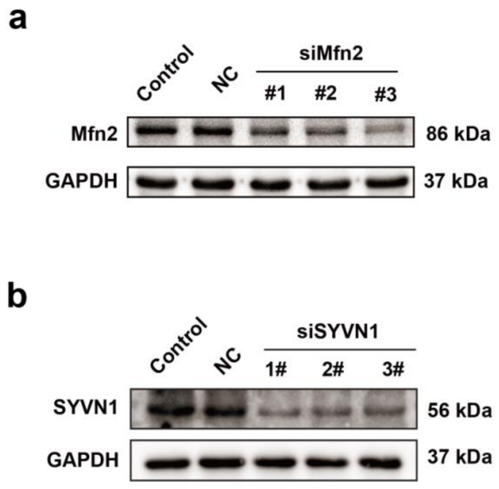

Supplementary Fig. S7. The efficacy of siMfn2 and siSYVN1 in A549 cells**. a** Cells were transfected with siMfn2 or nonspecific control siRNA (50 nM) for 24 h, and Mfn2 protein level was measured by western blotting. **b** A549 cells were transfected with siSYVN1 or nonspecific control siRNA (50 nM) for 24 h, and Mfn2 protein expression was determined by western blotting.
