# Supplementary figures and images for: Novel insights into stress-induced susceptibility to influenza: corticosterone impacts interferon-β responses by Mfn2-mediated ubiquitin degradation of MAVS
Source: Signal Transduct Target Ther. 2020 Sep 18;5:202. doi: 10.1038/s41392-020-00238-z (PMC7499204; doi:10.1038/s41392-020-00238-z)

**a**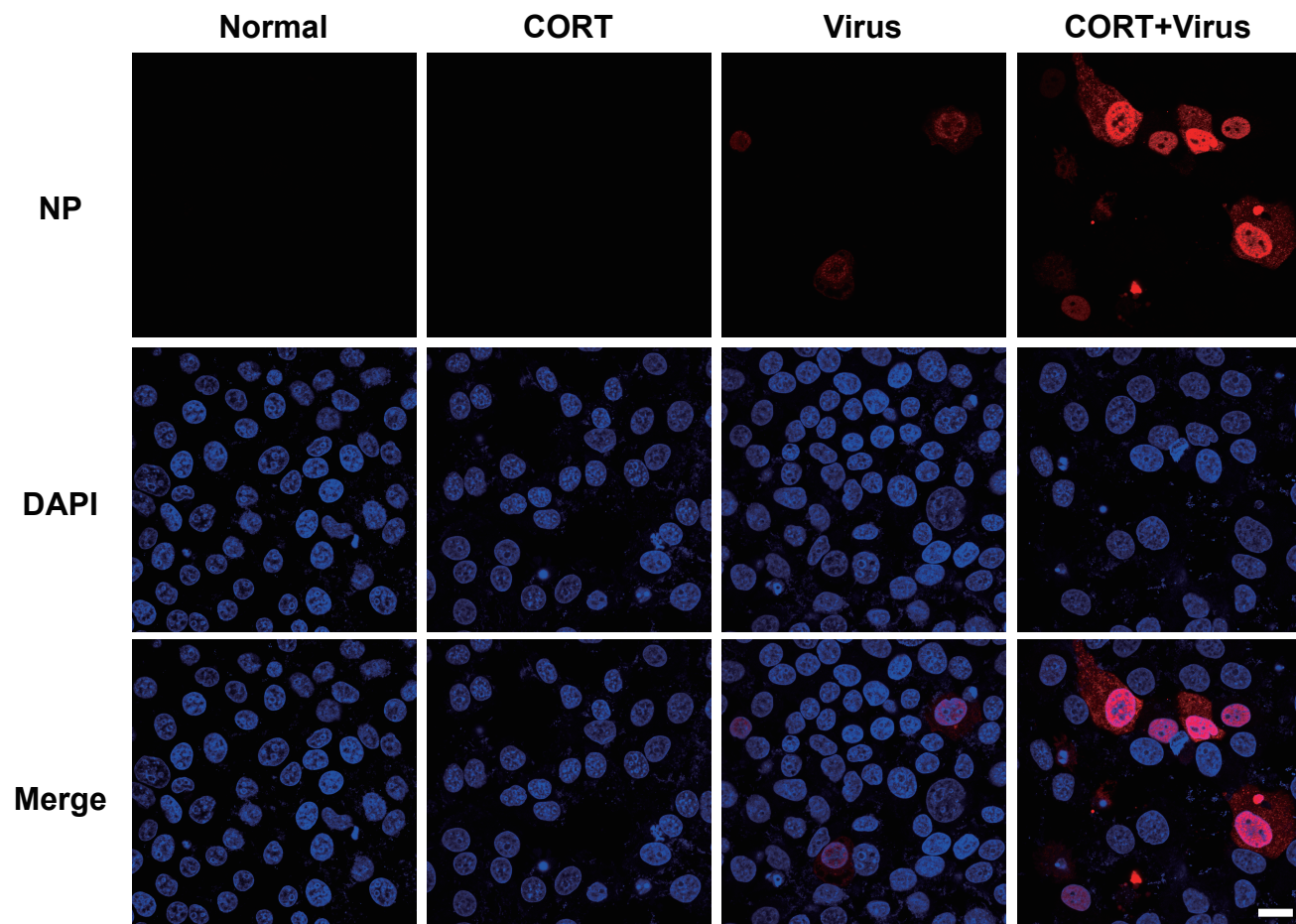**b**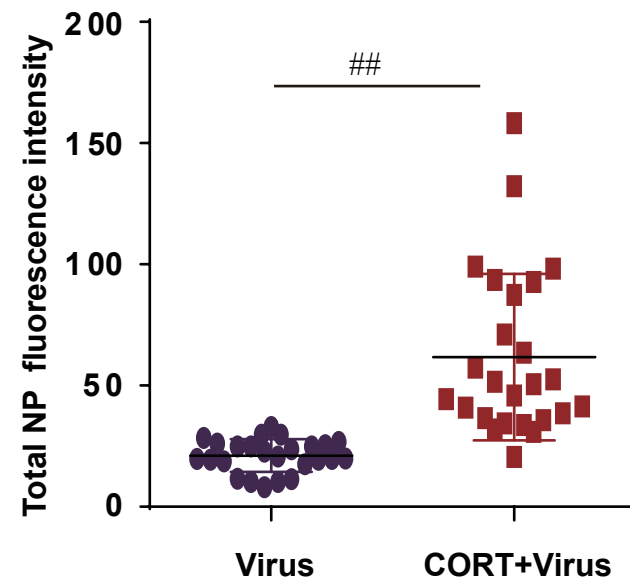

Supplement: Supplementary file 2 — Supplementary Fig. S1 [file 41392_2020_238_MOESM2_ESM.pdf]

**a**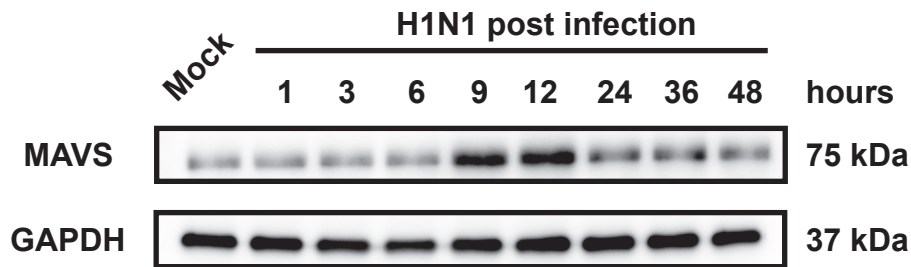**b**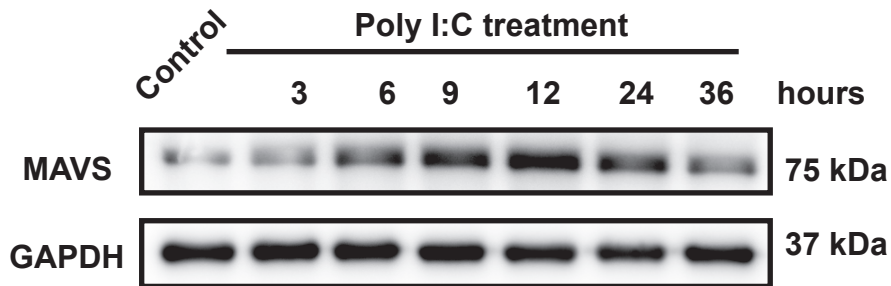

Supplement: Supplementary file 3 — Supplementary Fig. S2 [file 41392_2020_238_MOESM3_ESM.pdf]

**a**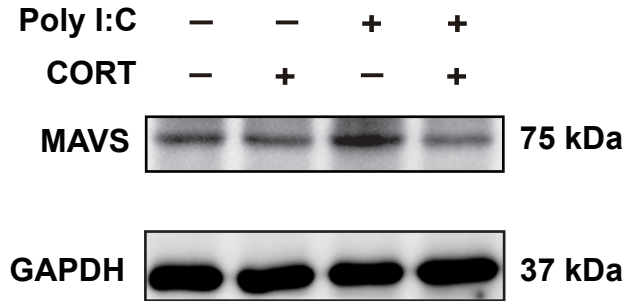**b**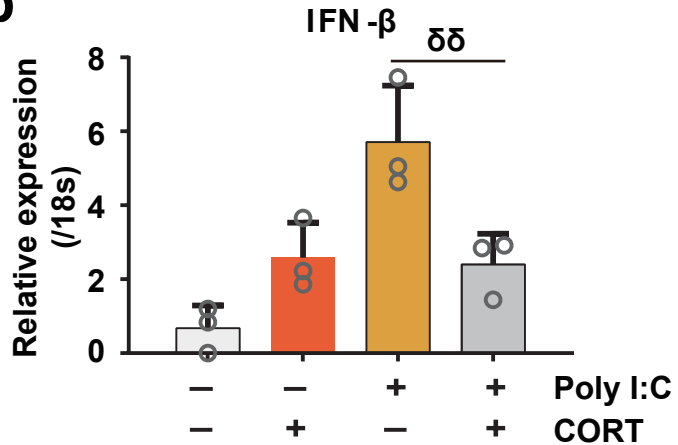

Supplement: Supplementary file 4 — Supplementary Fig. S3 [file 41392_2020_238_MOESM4_ESM.pdf]

**a**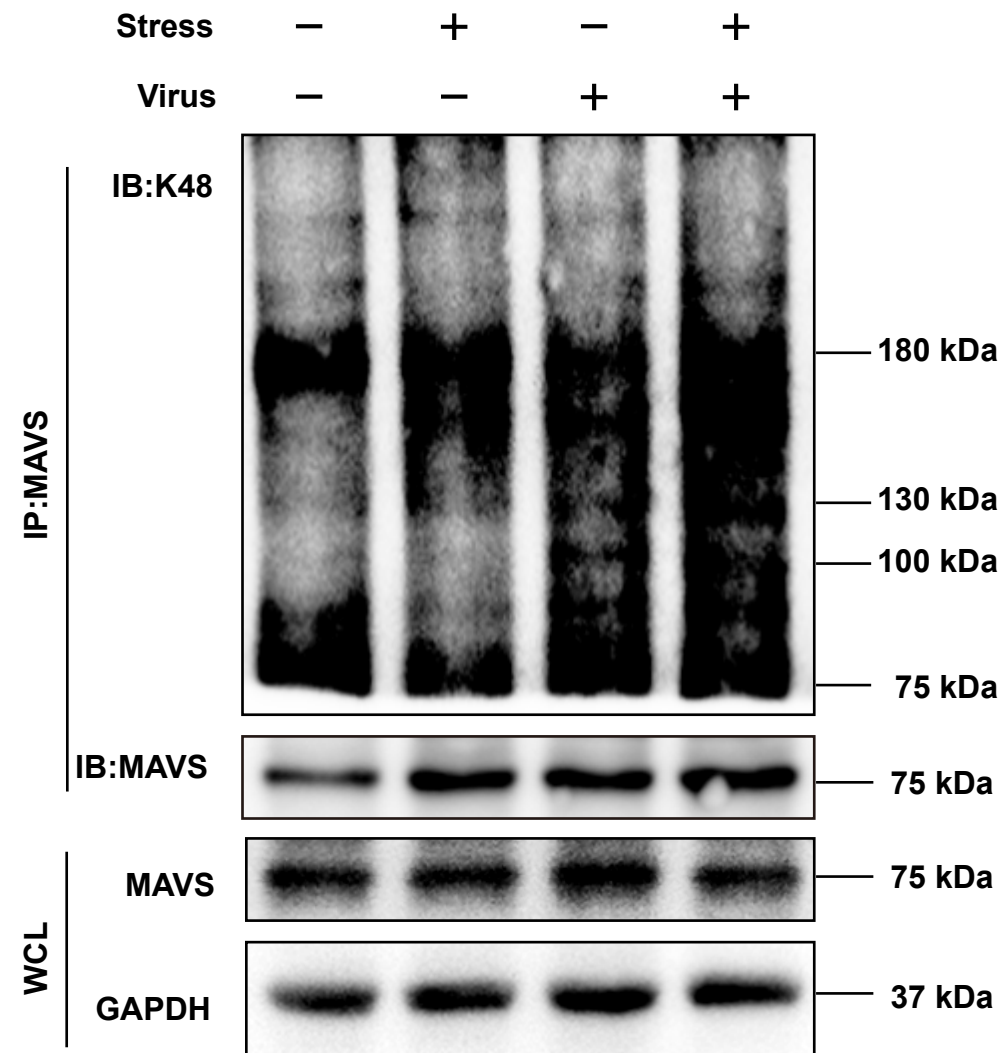**b**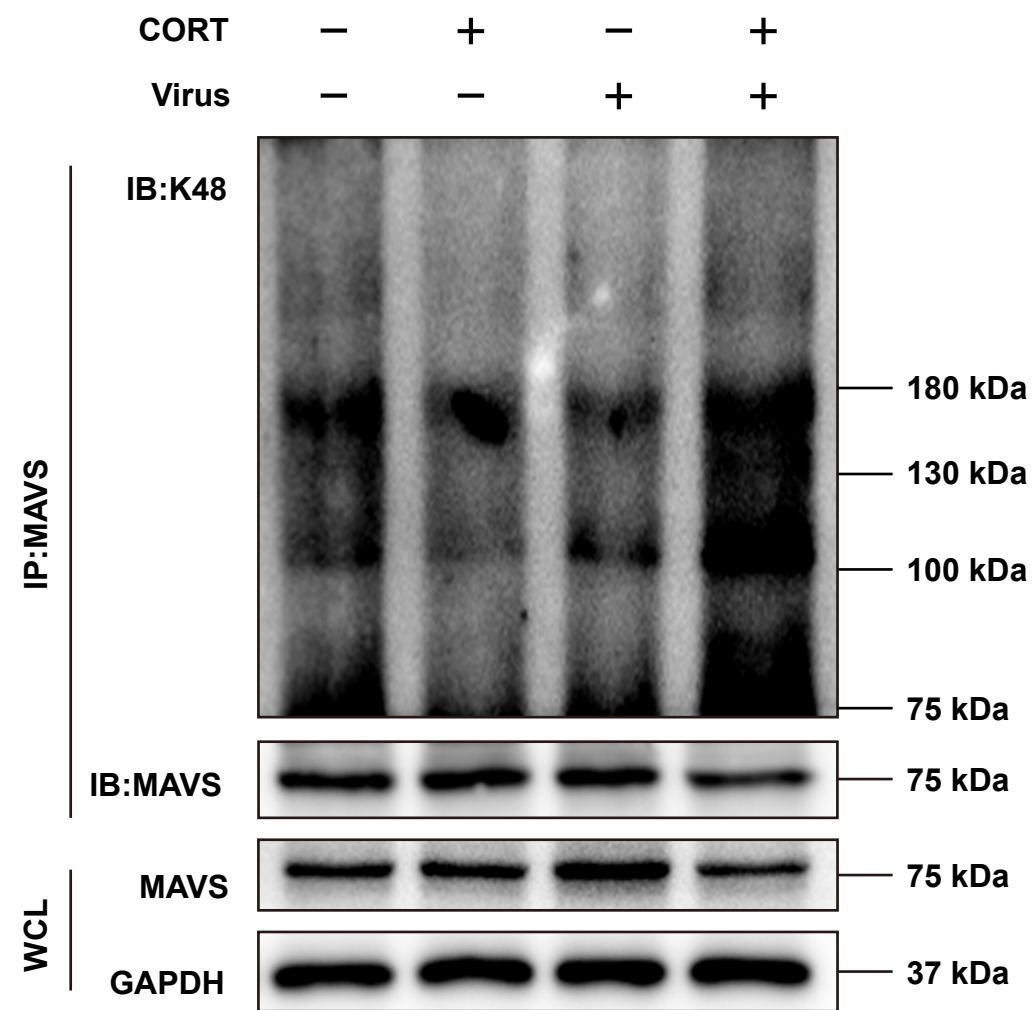

Supplement: Supplementary file 5 — Supplementary Fig. S4 [file 41392_2020_238_MOESM5_ESM.pdf]

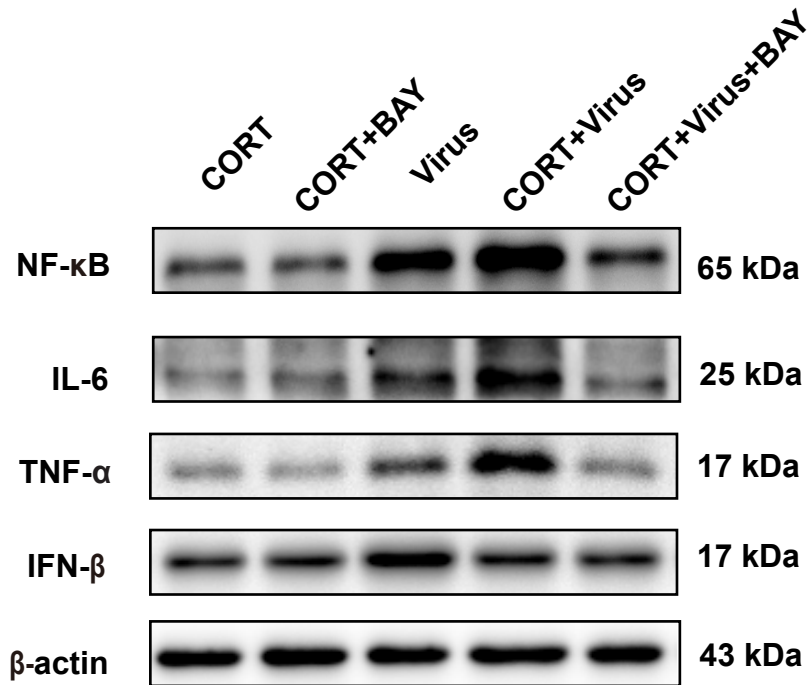

Supplement: Supplementary file 6 — Supplementary Fig. S5 [file 41392_2020_238_MOESM6_ESM.pdf]

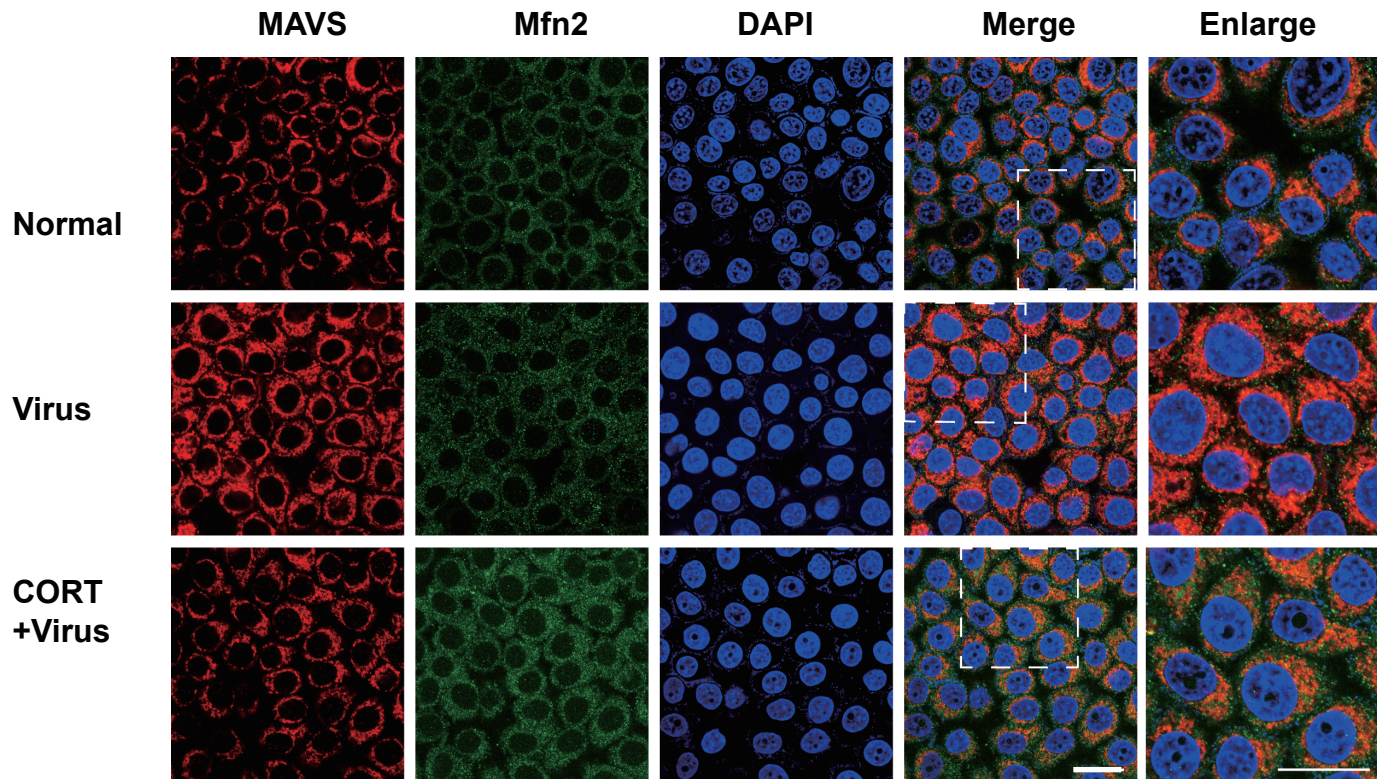

Supplement: Supplementary file 7 — Supplementary Fig. S6 [file 41392_2020_238_MOESM7_ESM.pdf]

**a**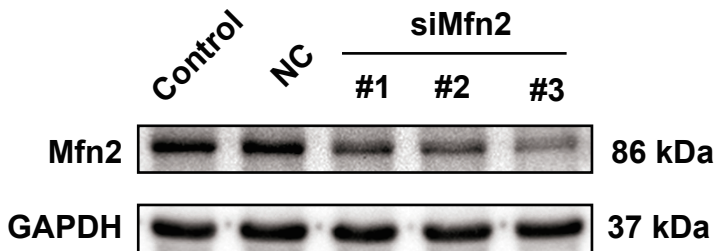**b**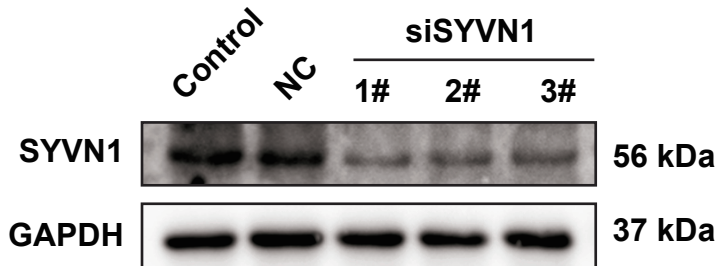

Supplement: Supplementary file 8 — Supplementary Fig. S7 [file 41392_2020_238_MOESM8_ESM.pdf]
